# Supplementary material for: Repetitive DNA Dynamics, Phylogenetic Relationships and Divergence Times in Andean Ctenomys (Rodentia: Ctenomyidae)
Source: Biology (Basel). 2025 Dec 12;14(12):1776. doi: 10.3390/biology14121776 (PMC12731067; doi:10.3390/biology14121776)
Supplement: Supplementary file 1 [file biology-14-01776-s001.zip › biology-3945796-supplementary.pdf]

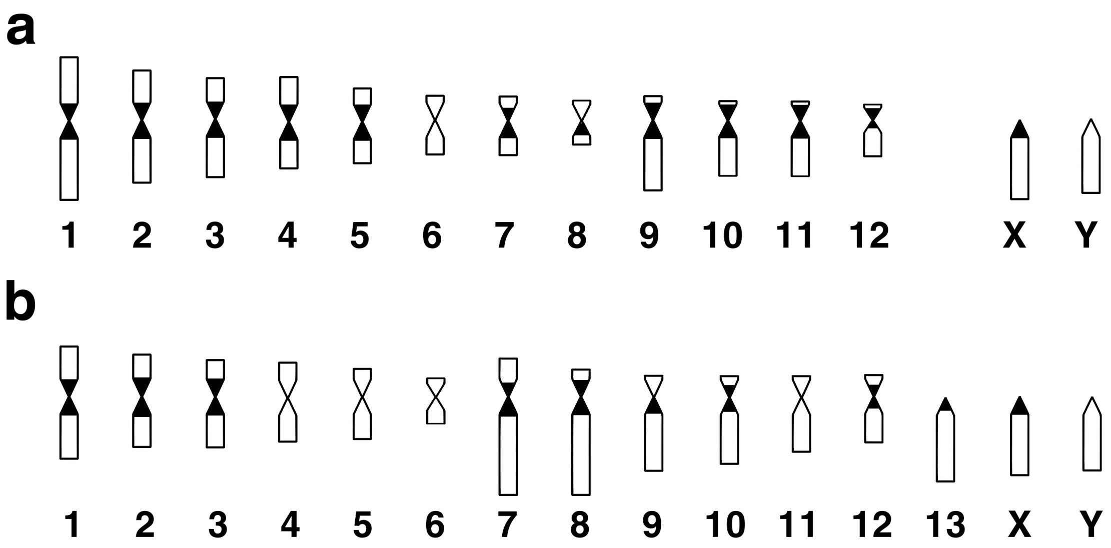

Figure S1. Idiograms of C-banding patterns in (**a**) *Ctenomys maulinus brunneus* and (**b**) *Ctenomys* sp., constructed following the descriptions of Gallardo [25].
